# Alcohol-Induced Alterations in the Vascular Basement Membrane in the *Substantia Nigra* of the Adult Human Brain

**DOI:** 10.3390/biomedicines10040830

**Published:** 2022-04-01

**Authors:** Sandra Skuja, Nityanand Jain, Marks Smirnovs, Modra Murovska

**Affiliations:** 1 Joint Laboratory of Electron Microscopy, Institute of Anatomy and Anthropology, Rīga Stradiņš University, LV-1010 Riga, Latvia; marks.smirnovs@rsu.lv; 2 Institute of Microbiology and Virology, Rīga Stradiņš University, LV-1007 Riga, Latvia; modra.murovska@rsu.lv

**Keywords:** vascular basement membrane, collagen-IV, laminin-111, fibronectin, substantia nigra, alcoholism, light microscopy, electron microscopy

## Abstract

The blood–brain barrier (BBB) represents a highly specialized interface that acts as the first line of defense against toxins. Herein, we investigated the structural and ultrastructural changes in the basement membrane (BM), which is responsible for maintaining the integrity of the BBB, in the context of chronic alcoholism. Human post-mortem tissues from the *Substantia Nigra* (SN) region were obtained from 44 individuals, then grouped into controls, age-matched alcoholics, and non-age-matched alcoholics and assessed using light and electron microscopy. We found significantly less CD31+ vessels in alcoholic groups compared to controls in both gray and white matter samples. Alcoholics showed increased expression levels of collagen-IV, laminin-111, and fibronectin, which were coupled with a loss of BM integrity in comparison with controls. The BM of the gray matter was found to be more disintegrated than the white matter in alcoholics, as demonstrated by the expression of both collagen-IV and laminin-111, thereby indicating a breakdown in the BM’s structural composition. Furthermore, we observed that the expression of fibronectin was upregulated in the BM of the white matter vasculature in both alcoholic groups compared to controls. Taken together, our findings highlight some sort of aggregation or clumping of BM proteins that occurs in response to chronic alcohol consumption.

## 1. Introduction

The blood–brain barrier (BBB) represents a highly specialized, organized, and dynamic interface that is involved in the regulation, homeostasis, and protection of the central nervous system (CNS). The barrier is composed of three different cell types, namely endotheliocytes, pericytes, and astrocytes, all of which interact with each other to maintain the function and integrity of the barrier [1,2]. However, it has been demonstrated that the BBB, in fact, is a part of a larger functional structure called the neurovascular unit (NVU), first described in 2001 by the Stroke Progress Review Group [3]. The different components of the NVU, including neurons and perivascular cells such as microglia and the basement membrane (BM), together with astrocytes, pericytes, and specialized unfenestrated endothelium, share complex and intimate associations that allow them to regulate blood flow along with the permeability of the BBB [3,4,5]. Under physiological conditions, the NVU prevents the entry of neurotoxic substances, blood cells, and pathogens into the brain parenchyma, allowing restricted transport of water, certain gases, ions, and molecules in and out of the CNS to maintain proper neuronal functioning [6,7,8,9]. 

However, in states of disease, trauma, or stress, the structural integrity of the BBB can be compromised, leading to barrier breakdown and dysfunction. Ultimately, this dysregulated BBB allows unrestricted movement of neurotoxins into the brain parenchyma, thereby attenuating and disrupting the signaling pathways involved in ensuring brain connectivity [10,11]. One such causative agent for BBB stress is alcohol (ethanol), a commonly abused psychoactive neurotoxin known to be associated with neuroinflammation and multiple neurodegenerative diseases, including Parkinson’s disease (PD), Alzheimer’s disease (AD), and epilepsy [12,13,14,15]. Previous studies have shown that alcohol and other similar drugs-of-abuse can cause a dose- and distribution-dependent dysfunction of the NVU by causing oxidative stress, altering protein expression, or dysregulating the endothelial tight junctions that bind the barrier [5,16,17,18,19].

The first line of contact, the endothelial cells in the BBB, are surrounded by a complex BM matrix comprising a three-dimensional network of a range of glycoproteins that include collagen-IV, laminin, and fibronectin [20]. These extracellular matrix (ECM) glycoproteins are mainly synthesized by the perivascular cells [21,22,23]. Collagen-IV is the most abundant fibrous protein in the BM and is secreted by endothelial cells, astrocytes, and pericytes. It plays an essential role in providing structural strength and integrity to the ECM by retaining laminin and other ECM proteins [24,25]. Laminins are the most abundant non-collagenous BM proteins. They are trimeric molecules consisting of α, β, and γ subunits. Out of the 16 possible different laminin isoforms, two isoforms, laminin-111 and laminin-211 (α1β1γ1 and α2β1γ1, respectively), are unique to the BBB and are produced by perivascular astrocytic cells [23]. These astrocytic laminins play a crucial role in maintaining BBB integrity and regulating pericyte function and astrocytic polarity [23]. Fibronectin is a disulfide-linked dimer secreted by endothelial cells, astrocytes, and pericytes [26]. Along with collagen-IV, fibronectin provides a structural support to the BBB. It mediates cell–cell attachment and functions to provide the organization of the ECM. Additionally, it stimulates the proliferation and survival of brain capillary endothelial cells in vitro [27]. 

In previous studies, it has been shown that the thicknesses of the glycoproteins containing BM ranges from 20 to 200 nm [21,28]. However, under pathological conditions such as AD and cerebral amyloid angiopathies, the BM thickness has been reported to increase, as evident from studies involving various animal models, human stroke tissue, and aging brain tissue [24,29,30,31,32,33]. In the AD brain, the degenerating endothelium has been postulated to cause disintegration of the cerebral BM, whilst an imbalance in lipid metabolism has been postulated as the cause of age-related lipid accumulation in the nearby end-feet of astrocytes, which leads to structural alterations in the BM [34,35]. In addition, various NVU-related factors such as activated matrix metalloproteinases, secreted inflammatory cytokines, extravasation of immune cells, and pathogens can play significant roles in the alteration and disruption of the BBB [34].

As part of the midbrain, the *Substantia Nigra* (SN) is located posteriorly to the *crus cerebri* fibers and morphologically divided into the dopaminergic *Pars Compacta* (SNpc) and inhibitory gamma-aminobutyric acid-containing (or GABAergic) *Pars Reticulata* (SNpr) [36,37,38]. The SN region plays an important role in a wide range of physiological processes, including movement control, learning, developing substance dependence, reward-seeking, and cognitive functioning [39,40,41,42]. Previous studies have shown the critical role of the SN region in addiction development or alcohol consumption processes, especially in correlation with other neurodegenerative disorders, including AD and PD [14,43]. Alcoholic beverages are rich in β-carbolines and their derivatives, which upon in vivo metabolization, form compounds resembling 1-methyl-4-phenylpyridinium ions (MPP+), neurotoxicants involved in the pathogenesis of idiopathic PD [44]. Additionally, in a number of aging-related and neurodegenerative diseases, it has been shown that there are significant changes in the microvascular length, tortuosity, and diameter in a region-specific manner [45]. At the same time, very limited data have been reported regarding the alcohol-specific effects on the network and density of the microvasculature, as well as the effects on the structural integrity and thickness of the BM and expression of ECM proteins in the human brain.

All of the above-mentioned findings highlight the role of the BBB in the SN in maintaining and preserving the SN microarchitecture. Since little is known about the disruptive role that alcohol can play in altering the BBB composition of the SN region, in the present study, we investigated the structural and ultrastructural changes in the BM matrix glycoproteins in the SN region, which are responsible for maintaining the integrity of the BBB in the context of chronic alcoholism.

## 2. Materials and Methods

### 2.1. Human Autopsy Brain Tissue Collection 

The present study included 44 individual brain autopsies as described previously (Supplementary Table S1) [46]. Briefly, 44 individuals were subdivided into three groups—controls (group A; 13 individuals, median age 31 ± 6.79 years), age-matched young alcoholics (group B; 13 individuals, median age 31 ± 4.85 years), and non-age-matched chronic alcoholics (group C; 18 individuals, median age 49.5 ± 8.66 years). The inclusion of the age-matched young alcoholics (group B) allowed us to exclude age-related effects in the present study.

The brain tissue was collected retrospectively (2007–2012) from the Latvian State Center for Forensic Medical Examination and preserved in paraffin blocks using standard laboratory techniques [46]. The protocol for the present study was approved by the Research Ethics Committee of Rīga Stradiņš University (Decision No. 6-1/12/9), dated 26 November 2020, per the provisions of the Declaration of Helsinki.

### 2.2. Immunohistochemistry Reactions

Formalin-fixed paraffin-embedded (FFPE) brain tissue samples from the *Substantia Nigra* (SN) region were used in the present study. Consecutive sections of 4–5 μm were used for histopathological and immunohistochemical (IHC) evaluation. FPPE samples were deparaffinized and dehydrated, followed by blocking of endogenous peroxidase activity using 3% H_2_O_2_ in methanol [46]. Sections were boiled in citrate buffer (pH 6) for retrieval of the antigens followed by incubation with primary antibodies (Table 1). 

Anti-CD31 antibody, a commonly used immunomarker, was used for the detection of endothelial cells [47]. Collagen-IV, a major constituent of the BM, was detected using anti-collagen type IV antibody [48]. Anti-laminin-111 antibody was used to determine the alpha 1, beta 1, and gamma 1 subunits found in the BM of CNS blood vessels [48]. Anti-fibronectin antibody was used for the determination of fibronectin in the BM [30].

Primary antibody amplification and visualization were performed via the HiDef Detection^TM^ HRP Polymer system (catalogue no: 954D-30; Cell Marque, Rocklin, CA, USA) and 3,3′ diaminobenzidine (DAB) tetrahydrochloride kit (catalogue no: 957D-30; DAB+ Chromogen and DAB+ Substrate buffer, Cell Marque, Rocklin, CA, USA) or using the HiDef Detection™ Alkaline Phosphatase Mouse/Rabbit Polymer System (catalogue no: 962D-30; Cell Marque, Rocklin, CA, USA) and Permanent Red Chromogen Kit (catalogue no: 960D-2; Cell Marque, Rocklin, CA, USA). Counterstaining of the sections was performed with Mayer’s hematoxylin, followed by series of washing, dehydration, clearing, and mounting in polystyrene. Visualization of brown-stained (DAB chromogen) or red-stained (red chromogen) structures was considered a positive reaction for respective IHC antibodies. For negative controls, IHC samples were stained with PBS (phosphate-buffered saline) solution. Microphotographs were collected using a Leitz bright-field microscope and DFC 450C digital camera (Leica-Leitz DMRB; Wetzlar, Germany), along with a Glissando Slide Scanner (Objective Imaging Ltd., Cambridge, UK). Additional measurements of tissue markers and their spatial distribution were obtained using Aperio ImageScope program v12.2.2.5015, Leica Biosystems, Chicago, IL, USA. 

### 2.3. Immunofluorescence Reactions

Immunofluorescence (IF) was performed by immunostaining samples with primary antibodies, followed by washing with PBS buffer. Fluorescent secondary antibodies (goat anti-mouse IgG (H + L) antibody, Alexa Fluor^®^ 488 conjugate (catalogue no. A1100; Thermo Fisher Scientific, Invitrogen, Waltham, MA USA, 1:300)) were used. Counterstaining was performed with 4’,6-diamidino-2-phenylindole (DAPI) followed by co-labeling with Prolong Gold and DAPI. Visualization of green-stained (fluorescent dyed) structures was considered a positive reaction. Confocal microscope Eclipse Ti-E (Nikon, Brighton, MI, USA) was used to capture digital images. 

The immunofluorescence intensity was measured for each of the three basement membrane proteins (collagen-IV, laminin-111, and fibronectin) using ImageJ software (ImageJ version 1.53p, U. S. National Institutes of Health, Bethesda, ML, USA). The corrected total cell fluorescence (CTCF) method was used to adjust the IF intensity for background noise correction. For CTCF calculations, thirty microvessels per protein were randomly chosen (ten per grade) and visualized at 1000× magnification (Supplementary Figure S1). Negative controls for IHC and IF are shown in Supplementary Figure S2. 

### 2.4. Transmission (TEM) and Scanning (SEM) Electron Microscopy

TEM and SEM were used for the ultrastructural examination of the tissue samples. Tissue processing was performed in accordance with routine laboratory protocols by fixing samples in 2.5% glutaraldehyde. Post-fixation was performed using osmium tetroxide (OsO_4_). This was followed by dehydration and embedment in epoxy resin (Sigma-Aldrich, Buchs, Switzerland). LKB ultramicrotome was used to obtain semi-thin sections measuring 1 μm, which were then stained with 1% toluidine blue for structural analysis in the light microscope. Next, ultra-thin (60 nm) sections were obtained, collected on formvar-coated 200-mesh nickel grids, and stained with 2% uranyl acetate and lead citrate. Sections were examined by JEM 1011 (JEOL, Akishima, Tokyo, Japan). For the ultrastructural analysis, thirty microvessels with a transverse profile were randomly chosen and visualized at 12,000× magnification equally from each group. The thickness of the BM was determined by taking 10 measurements per vessel in the ImageJ program.

Tissue samples underwent dehydration using a series of graded solutions of acetone for SEM analysis, and then dried with liquid CO_2_ using the critical point method (E3000 drying device, Agar Scientific, Stansted, UK). Samples were then covered with a gold layer and examined using JSM-6490LV (JEOL, Akishima, Tokyo, Japan) at an accelerating voltage of 25 kV and at magnification range of 5000–10,000×.

### 2.5. Scoring System and Statistical Analysis

**Figure 1:**
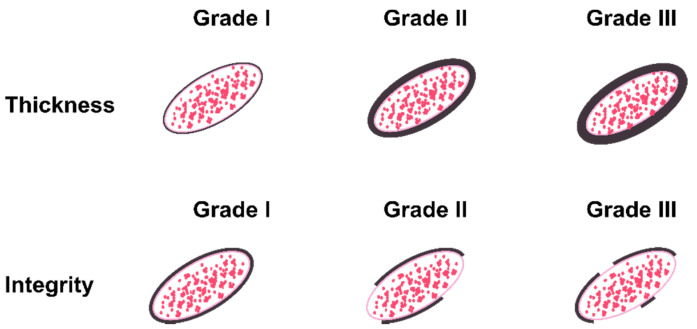
Semi-quantitative grading scale used for analysis of the thickness and integrity of the vascular BM. Red dots symbolize erythrocytes. Each vessel was analyzed for both these parameters individually in five random visual fields per region, per sample for both gray and white matter. Grade I represents normal vessels. Grade II represents moderately damaged vessels. Grade III represents severely damaged vessels.

A quantitative scoring system was used to assess the positively stained microvessels (using CD31 antibody) by two independent observers at 400× magnification in 10 visual fields per region, per sample. For the analysis of the endothelial BM architecture, a semi-quantitative grading scale was developed (Figure 1). Two different parameters were registered for each immunohistochemically positive protein (collagen IV, laminin-111, and fibronectin) in the BM microvessels, namely thickness and integrity. The scale was designed based on the color signal intensity with low to high expression of BM proteins. For BM thickness: Grade I—normal thickness (defined as a visually detectable baseline or minimal quantity of immunoreaction products); grade II—moderately increased thickness; grade III—highly increased thickness. For BM integrity: Grade I—no visible changes; grade II—mildly to moderately disrupted BM; grade III—highly disrupted BM. All vessels in five random visual fields per region, per sample were evaluated by two independent observers to assess thickness and integrity. Approximately 8000–10,000 microvessels were investigated per immunoreaction for thickness and integrity. 

The collected data was stored and digitalized in MS Excel (Microsoft Office 365). For CD31, the data distribution was checked using the Shapiro–Wilk test for normality (*p* < 0.05 indicates a violation of the normality). Non-parametric tests were used due to violation of normality. Kruskal–Wallis ANOVA was used for inter-group analysis with appropriate post hoc tests and Bonferroni correction. Intra-group analysis was performed using related-samples Wilcoxon signed rank test [46]. 

Due to the Likert-type nature of the semi-quantitative scoring system, for other immunohistochemical markers (collagen-IV, laminin-111, and fibronectin), Kruskal–Wallis ANOVA was used for inter-group analysis and related-samples Wilcoxon signed rank test was used for intra-group analysis for a location shift of distribution. For the purpose of visualization, the weighted average of the grades assigned was calculated and used. Correlation analysis was performed using Spearman’s Rho. Statistical significance was set as *p* < 0.05. All analyses were perfomed using SPSS (IBM Corp. Released 2020; IBM SPSS Statistics for Windows 10, Version 27.0; Armonk, NY, USA: IBM Corp). Graphical representations were performed in R studio and MS Excel.

## 3. Results

### 3.1. Alcoholics Showed Significantly Less CD31+ Vessels Than Controls in Both Gray and White Matter

**Figure 2:**
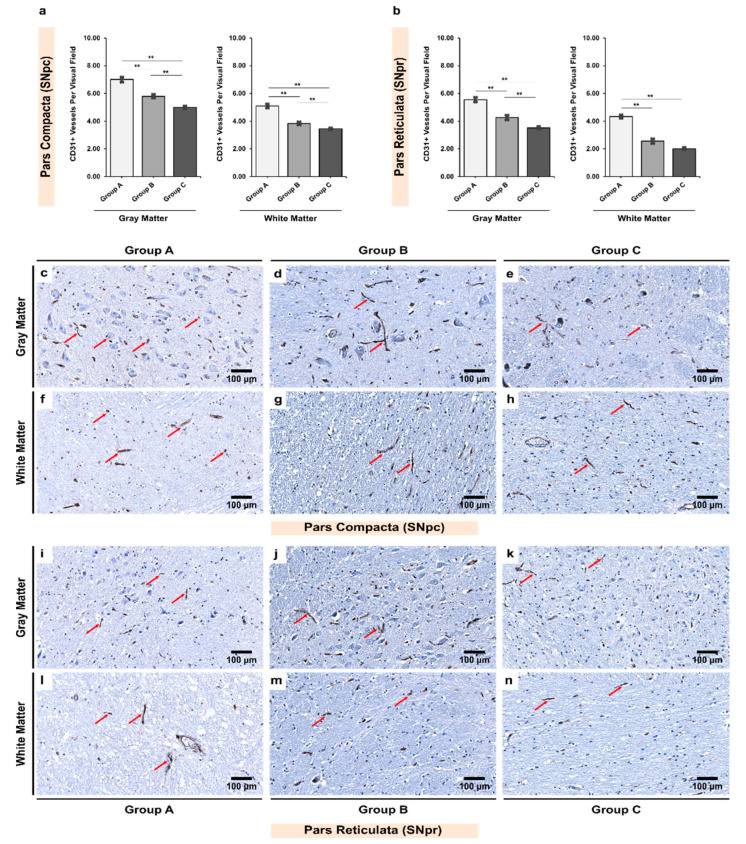
Inter-group analysis of CD31+ vessels per visual field in the (**a**) *Pars Compacta* (SNpc) and (**b**) *Pars Reticulata* (SNpr) for the three studied groups in both gray and white matter. The bar plots indicate the average number of CD31+ vessels ± S.E. (Standard Error) seen per visual field; ** indicates a significant difference between the groups (*p* < 0.05 with Bonferroni correction is considered as significant). Distribution of CD31+ vessels per visual field in (**c**,**f**,**i**,**l**) group A (controls), (**d**,**g**,**j**,**m**) group B (age-matched alcoholics), and (**e**,**h**,**k**,**n**) group C (non-age-matched alcoholics). Red arrows indicate CD31+ vessels. Original magnification, 200×. Scale bars, 100 µm.

We found significantly more CD31+ vessels in the gray matter than in the white matter for both the SNpc and SNpr in all three groups (*p* < 0.001; Table 2). Further, a significant decrease in the number of vessels was noted in age-matched alcoholics (group B) when compared with controls (group A; Figure 2). There was further a significant decrease between age-matched and non-age-matched alcoholics (group B vs. C).

### 3.2. Alcoholics Showed Significant Increases in Collagen-IV Expression Coupled with Significant Losses of Vessel Integrity in Both Gray and White Matter

**Figure 3:**
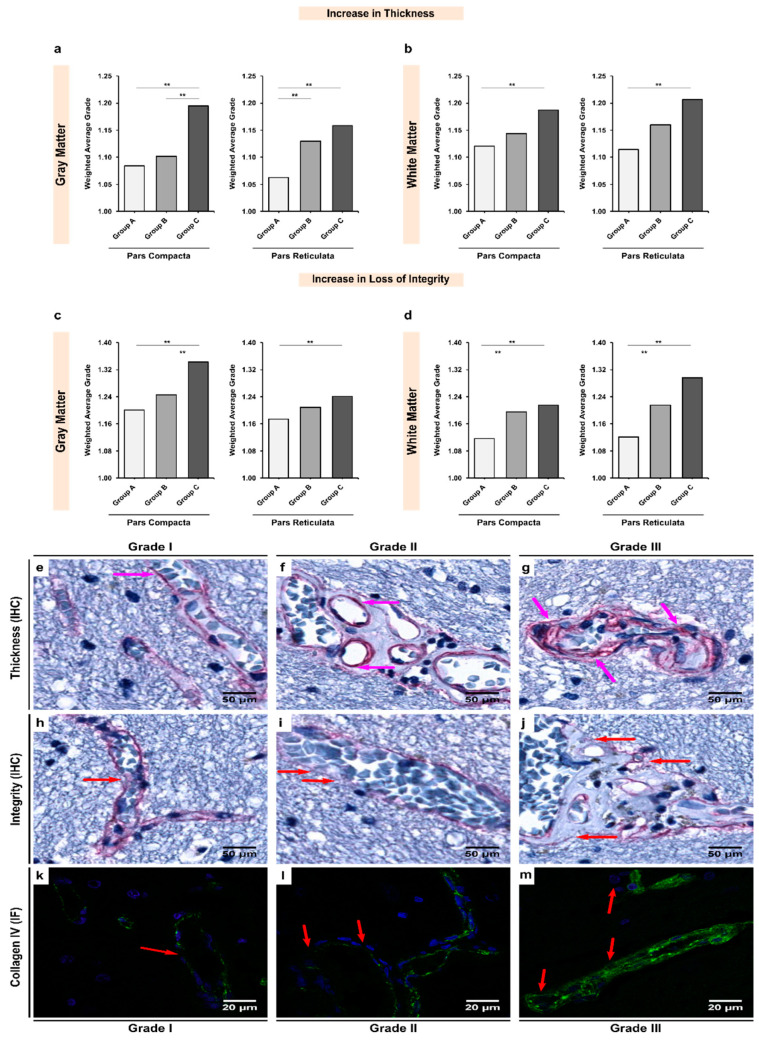
Inter-group analysis of the increase in (**a**,**b**) thickness and (**c**,**d**) loss of integrity of the BM based on the expression of collagen-IV in the microvessels in the (**a,c**) gray matter and (**b**,**d**) white matter of the SN region. Group A represent controls, group B represents young alcoholics, whilst group C represents chronic alcoholics. The bar plots indicate the weighted average grading of all the visualized blood vessels in each group; ** indicates a significant difference between the groups (*p* < 0.05 with Bonferroni correction is considered as significant). Representative photomicrographs showing the different grades of BM thickness and integrity based on the expression of collagen-IV as visualized in the brain tissue material using immunohistochemistry (IHC) and immunofluorescence (IF) as follows: (**e**,**h**,**k**) grade I vessel with normal (baseline) thickness and unchanged integrity; (**f**,**i**,**l**) grade II vessel with moderate thickness and damaged integrity; (**g**,**j**,**m**) grade III vessel with extremely thickened and split BM. The pink and red arrows show the extent of thickening and loss of integrity as visualized using IHC and IF, respectively. In IF, green color shows the collagen-IV protein, whilst blue color shows DAPI-stained nuclei. Original magnification (IHC), 400×. Scale bars, 50 μm. Original magnification (IF), 1000×. Scale bars, 20 μm.

The expression of collagen-IV in the BM of white matter microvessels appeared to be more abundant when compared with gray matter microvessels in both the SNpc (insignificant; *p* = 0.072; Table 3) and SNpr (significant; *p* = 0.002; Table 3) in the control group. Overall, we observed that alcohol exposure led to a significant increase in the BM thickening due to the over-expression of collagen-IV (Figure 3). In the SNpc, there was a significant increase in the collagen-IV expression between chronic alcoholics and controls in both gray and white matter (groups A–C; *p* < 0.001 and 0.009, respectively). Similar difference was noted in the SNpr gray and white matter (groups A–C; *p* < 0.001 and 0.001, respectively). Additionally, the SNpc seemed to be more affected by alcohol-mediated collagen-IV over-expression (Supplementary Table S2). 

In terms of the integrity of the collagen-IV in the BM of vasculature, gray matter presented with more fragile BM than white matter in both the SNpc and SNpr of controls (*p* < 0.001; Table 4). In line with our expectations, alcoholism led to a significant disruption of the BM’s integrity, especially more in the white matter (Supplementary Table S3 and Figure 3). In both the SNpc and SNpr white matter, there were significant differences noted between controls and age-matched alcoholics (groups A and B; *p* = 0.008 and 0.029, respectively) and controls and chronic alcoholics (groups A–C; *p* < 0.001 and 0.002, respectively). In the gray matter, chronic alcoholics showed a significantly more disrupted BM in both the SNpc and SNpr (groups A–C; *p* = 0.012 and *p* < 0.001, respectively).

### 3.3. Expression of Laminin-111 Showed Significant Increases in Alcoholics Coupled with Significant Changes in the Vessel Integrity in Both Gray and White Matter

**Figure 4:**
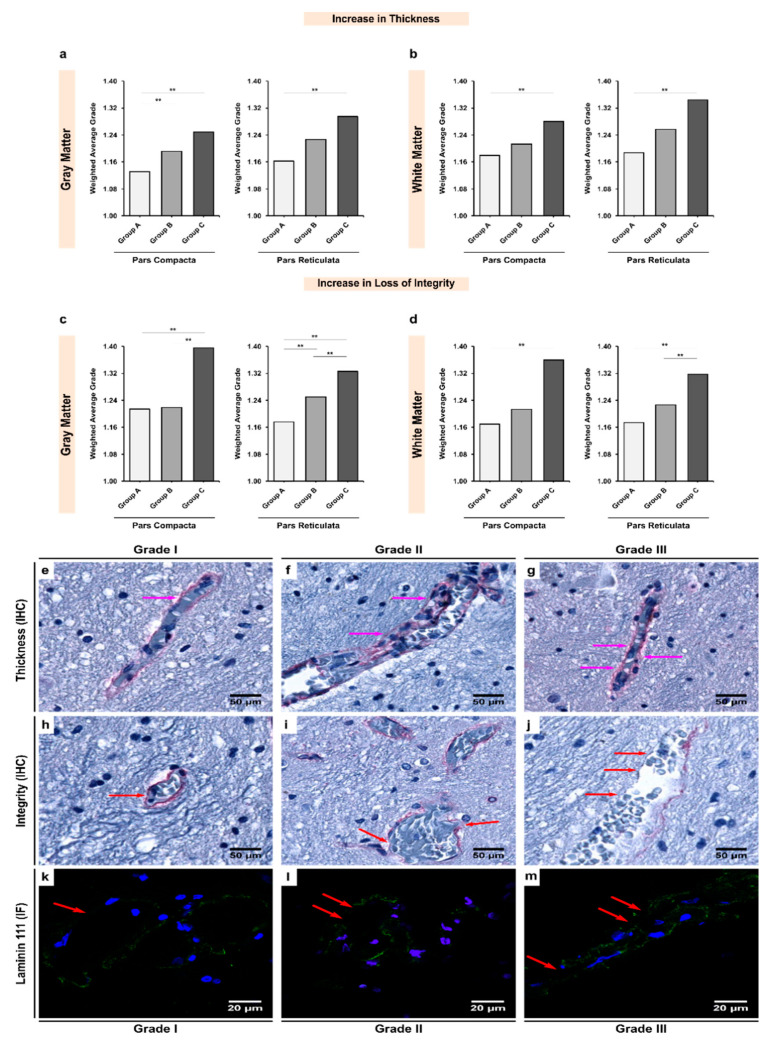
Inter-group analysis of the increase in (**a**,**b**) thickness and (**c**,**d**) loss of integrity of the BM based on expression of laminin-111 in the microvessels in the (**a**,**c**) gray matter and (**b**,**d**) white matter of the SN region. Group A represent controls, group B represents young alcoholics, whilst group C represents chronic alcoholics. The bar plots indicate the weighted average grading of all the visualized blood vessels in each group; ** indicates a significant difference between the groups (*p* < 0.05 with Bonferroni correction is considered as significant). Representative photomicrographs showing the different grades of thickness and integrity of BM based on expression of laminin-111 as visualized in the brain tissue material using immunohistochemistry (IHC) and immunofluorescence (IF) as follows: (**e**,**h**,**k**) grade I vessel with normal thickness and unchanged integrity; (**f**,**i**,**l**) grade II vessel with moderate thickness and damaged integrity; (**g**,**j**,**m**) grade III vessel with extremely thickened and split BM. The pink and red arrows show the extent of thickening and loss of integrity as visualized using IHC and IF, respectively. In IF, green color shows the laminin-111 protein, whilst blue color shows DAPI-stained nuclei. Original magnification (IHC), 400×. Scale bars, 50 μm. Original magnification (IF), 1000×. Scale bars, 20 μm.

Although a stronger expression of laminin-111 was observed in the BM of white matter microvessels, the difference when compared with gray matter microvessels remained insignificant across all three groups (*p* > 0.05; Table 5). Inter-group analysis revealed that alcohol exposure led to a significant increase in laminin-111-mediated BM thickening, in line with the changes observed for collagen-IV (Figure 4). A significant increase in laminin-111 expression was noted between chronic alcoholics and controls in both gray and white matter of the SNpc (groups A–C; *p* < 0.001 and 0.005, respectively) and SNpr (groups A–C; *p* = 0.001 for both regions). However, unlike collagen-IV, it was the SNpr that seemed to be more affected by alcohol-mediated laminin-111 overexpression (Supplementary Table S2).

Similar to the results for collagen-IV expression, the BM of the gray matter vasculature was found to be more fragile and disintegrated, as also demonstrated by the expression of laminin-111, potentially indicating a complete breakdown in the BM’s structural composition due to chronic exposure to alcohol (Table 4). These changes were more prominent in the gray matter than white matter. This shows that possibly the gray matter vasculature is more sensitive to laminin-111-mediated changes whilst the white matter vasculature is more sensitive to collagen-IV mediated changes (Table 3, Table 4, Table 5 and Table 6; Supplementary Tables S2 and S3). In both the SNpc and SNpr gray matter, we observed significant laminin-111 disruption in chronic alcoholics when compared with controls (groups A–C; *p* < 0.001 and 0.014, respectively). Additionally, there were significant differences noted between age-matched alcoholics and chronic alcoholics (Groups B and C; *p* = 0.001 and 0.013, respectively), indicating that laminin-111 integrity may be linked to consumption patterns of alcohol.

### 3.4. Expression of Fibronectin Was Significantly Upregulated in Alcoholics, Which Was Coupled with Significant Loss of Structural Integrity in Both Gray and White Matter

**Figure 5:**
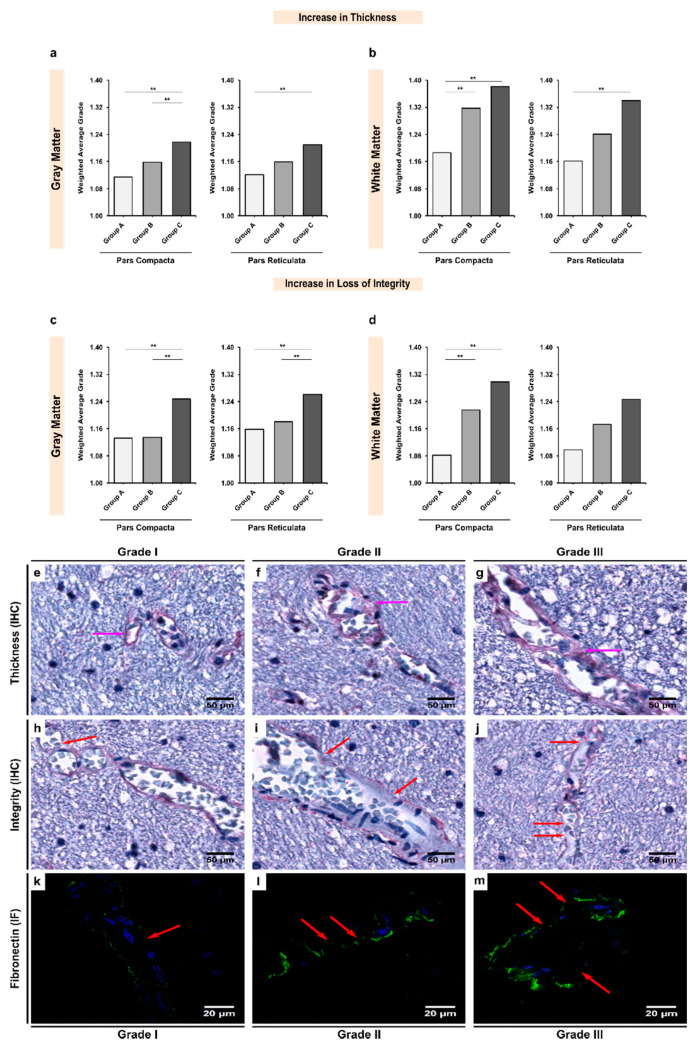
Inter-group analysis of the increase in (**a**,**b**) thickness and (**c**,**d**) loss of integrity of the BM based on the expression of fibronectin in the microvessels in the (**a**,**c**) gray matter and (**b**,**d**) white matter of the SN region. Group A represent controls, group B represents young alcoholics, whilst group C represents chronic alcoholics. The bar plots indicate the weighted average grading of all the visualized blood vessels in each group; ** indicates a significant difference between the groups (*p* < 0.05 with Bonferroni correction is considered as significant). Representative photomicrographs showing the different grades of BM thickness and integrity based on expression of fibronectin as visualized in the brain tissue material using immunohistochemistry (IHC) and immunofluorescence (IF) as follows: (**e**,**h**,**k**) grade I vessel with normal (baseline) thickness and unchanged integrity; (**f**,**i**,**l**) grade II vessel with a moderate thickness and damaged integrity; (**g**,**j**,**m**) grade III vessel with extremely thickened and split BM. The pink and red arrows show the extent of thickening and loss of integrity as visualized using IHC and IF, respectively. In IF, green color shows the fibronectin protein whilst blue color shows DAPI-stained nuclei. Original magnification (IHC), 400×. Scale bars, 50 μm. Original magnification (IF), 1000×. Scale bars, 20 μm.

We observed that fibronectin was significantly more expressed in the BM of the white matter vasculature in both the SNpc and SNpr across all three groups (*p* < 0.05; Table 7), except in the SNpr of the controls, where the difference was found to be insignificant (*p* = 0.923; Table 7). Furthermore, like the other two BM glycoproteins, expression of fibronectin was also upregulated due to alcohol exposure (Figure 5), thereby contributing to the thickening of the BM. In the SNpc, a significant increase in the expression of fibronectin was noted in both gray and white matter in chronic alcoholics when compared with controls (groups A–C; *p* < 0.001 and 0.036, respectively). In SNpr gray and white matter, a similar observation was made (groups A–C; *p* = 0.001 and 0.041, respectively). Similar to laminin-111, the SNpr seemed to be more affected by alcohol-mediated overexpression of fibronectin (Supplementary Table S2).

It is noteworthy that for fibronectin, it appeared that the BM of the vasculature was affected differently based on the region. In the SNpc the white matter vasculature was more affected, whilst in the SNpr it was the gray matter vasculature that was more affected in terms of the fibronectin structural integrity (Table 8). The underlying mechanisms and an explanation for such observations would be essential to investigate in future studies. In SNpc gray and white matter, there were significant differences between chronic alcoholics and controls (groups A-C; *p* < 0.001 and 0.006, respectively) and age-matched alcoholics (groups B and C; *p* = 0.001 and 0.003, respectively). In the SNpr, no significant changes were observed in the expression of fibronectin in the white matter vasculature, but a significant increase was noted in the gray matter vasculature (Table 8 and Supplementary Table S3). A significant disruption in fibronectin expression was noted in chronic alcoholics when compared with controls (groups A–C; *p* = 0.004) and age-matched alcoholics (groups B and C; *p* = 0.001).

### 3.5. Increases in the Thickness or Expression of BM Glycoproteins Were Negatively Correlated with the Integrity of the BM

Although the correlation analysis revealed significant associations, the strengths of the correlation factors between the expression levels of the three BM glycoproteins were negligible in all cases. This indicates that although though the expression patterns of glycoproteins are inter-related, the expression remains independent of the changes in the expression of the other two glycoproteins. In terms of the integrity of the gray matter vasculature, the expression of fibronectin was significantly correlated with the expression of collagen-IV and laminin-111 (ρ = 0.03 and 0.04, respectively; *p* = 0.040 and 0.006, respectively). In the BM of the white matter vasculature, only the expression of laminin-111 and fibronectin was significantly correlated (ρ = 0.06; *p* = 0.021). In terms of the thickness of the BM (overexpression of glycoproteins), the fibronectin expression in the gray matter vasculature correlated negatively and significantly with that of collagen-IV (ρ = −0.04; *p* = 0.005).

In terms of individual protein analysis, collagen-IV expression in both gray and white matter was found to be significantly and negatively correlated in terms of thickness and integrity of the glycoprotein (ρ = −0.08 and −0.07, respectively; *p* = 0.001). Similar observations were made for laminin-111 in both the gray (ρ = −0.11; *p* = 0.001) and white matter vasculature (ρ = −0.09; *p* = 0.001). Fibronectin also showed a similar trend in both the gray (ρ = −0.07; *p* = 0.038) and white matter vasculature (ρ = −0.06; *p* = 0.008). These findings indicate that the integrity of the BM decreases whilst the thickness or expression of the glycoproteins increases, thereby implying some sort of aggregation or clumping of the glycoproteins. Such structural alterations in the BM of the vasculature in the SN region require further investigation.

### 3.6. Ultrastructural Analysis of the Vascular Basement Membrane and BBB 

The integrity of the BM varied from a homogeneous appearance to pronounced multi-lamellar aspects. Using perpendicular measurements for the distance between the inner and outer edges of the BM, the detected thickness varied from 29.8 nm to 2406.7 nm, with an average thickness of the separate lamella of around 256 nm (Supplementary Table S4). Grade I vessels showed an average thickness of the BM of 107.2 ± 43.4 nm, whilst for grade II vessels the average thickness was 205.4 ± 115.9 nm. Grade III vessels showed an average thickness of around 433.9 ± 402.1 nm. 

**Figure 6:**
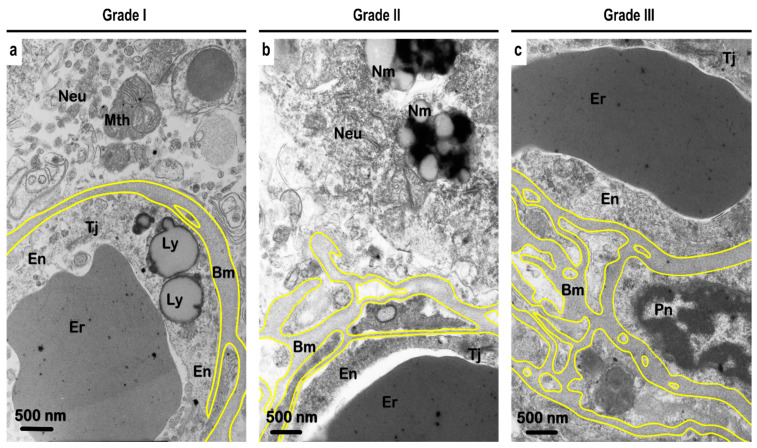
Representative transmission electron microscopy (TEM) micrographs of ultrastructural changes observed in different grades of blood vessels in the gray matter of predominantly (**a**) controls and (**b**,**c**) alcoholics. The yellow lines indicate the outer borderline of the basement membrane (Bm). (**a**) Accumulation of lipolysosomes in the cytoplasm of the endothelial cell. Homogenous and smooth BM can be seen. (**b**) Endothelial cells with tight junctions and nearby neuron containing neuromelanin is seen. The vessel has a lamellar BM. (**c**) Endothelial cells with tight junctions and pericyte with nucleus can be seen. The vessel shows splitting of the BM. Abbreviations: Neu, neuron; Er, erythrocyte; Pn, nucleus of pericyte; En, endothelial cell; Tj, tight junction; Mth, mitochondria; Nm, neuromelanin; Ly, lipolysosomes. Original magnification, 12,000×. Scale bars, 500 nm.

Apart from the various shapes, BM lamellae encircled the cytoplasm of the pericytes and the endothelial cells (Figure 6). The endothelial cells were characterized by a variably flattened shape and showed an electron-dense cytoplasm. Large lipid-containing lysosomes were frequently found inside the endotheliocytes. In some vessels, the tight junction complexes appeared swollen. Whilst a wavy nuclear envelope appearance was detected in both the endothelial cells and pericytes, nuclei showed peripheral and homogeneous clusters of heterochromatin. Pericytes showed an electron-dense cytoplasm with an expanded rough endoplasmic reticulum cisternae and variable mitochondria. Perivascular astrocytic foot processes often were swollen, showing an electron-lucid “empty” cytoplasm. At the same time, astrocyte processes that were diffused from the microvessels demonstrated large, swollen mitochondria and well-preserved cytoskeleton elements.

**Figure 7:**
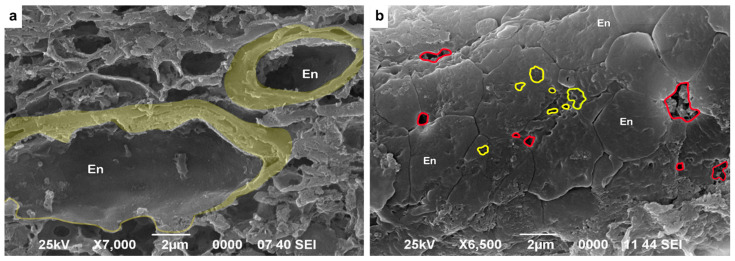
Representative scanning electron microscopy (SEM) micrographs of the vascular endothelium in the gray matter of predominantly (**a**) controls and (**b**) alcoholics. (**a**) Characteristic continuous endothelium (En) seen in the walls of SN microvessels in the lateral view. Original magnification, 7000×. (**b**) Ultrastructural changes observed in between neighboring endothelial cells on the luminal surface. Large paracellular pores (shown by a red line) and fenestrae (shown by a yellow line) can be seen on the luminal surfaces of endothelial cells. Original magnification, 6500×. Scale bars, 2 μm.

Interestingly, we also observed fenestrae on the surfaces of the endothelial cells. The fenestrae showed different shapes, ranging from an elongated shape to a more roundish shape, with an average size of around 500 nm in diameter. Additionally, we found large paracellular pores between the neighboring endothelial cells, which varied in size from around 500 nm to 2 µm (Figure 7).

## 4. Discussion

### 4.1. White Matter Has Significantly Fewer CD31+ Microvessels Than Gray Matter in Physiological Conditions

The SN region receives its blood supply from both the paramedian branches of the basilar artery and posterior cerebral artery providing a blood supply to the medial–caudal portion, as well as the anterior choroid artery providing blood supply to the most medial–superior part [49]. The rich blood supply to the region makes it extremely sensitive and more exposed to the alcohol in circulation [50]. The unique tree-like branching geometry of the vasculature in the SN region extends from the white matter to the gray matter, thereby causing the white matter to have less branched and more dilated microvessels whilst the gray matter possess more branched but narrower microvessels, in line with our findings from CD31+ observations (Table 2 and Figure 2). Similar distribution patterns of microvessels have been reported in other subcortical regions [51,52,53]. 

In a human brain autopsy study, the authors showed that the intraparenchymal vessels in the basal ganglia resembled long arterioles and long muscular arteries with no interdigitating arteriolar fields, and originated from a single source, thereby putting the region at increased risk of hypoperfusion and anoxia [54]. In fact, it is no coincidence that this region is the most frequent site for small lacunar infarcts and other degenerative vessel wall conditions [55] and is extremely sensitive to hypertensive and aging-related changes, with arteries forming twists, spirals, and loops [56].

A study in human fetuses by Ballabh et al. showed that both the % of blood vessel area and number of vessels per mm^2^ were higher in gray matter than the white matter in the frontal cortex, and the trend remained the same from as early as 16–20 gestational weeks [57]. In fact, the difference in vasculature density increased with increasing gestational weeks, with the authors finding that the gray matter vasculature started to expand in density and % from the 16th gestational week, whilst the white matter vasculature showed a similar phenomenon only close to gestational maturity after the 32nd gestational week [57]. It has been demonstrated that in comparison to the white matter, the basal ganglia and frontotemporal cortex of premature infants had more cerebral blood flow [58]. Similar observations have been reported using susceptibility contrast enhancement MRI techniques in mature infants and adult brains [59]. The results obtained in the present study confirm these previous findings, especially since these previous studies have used different antibodies to report their findings (we used the endothelial anti-CD31 antibody); whilst one study relied on anti-laminin antibody [57], the other study used anti-laminin, anti-collagen-IV, and anti-fibronectin antibodies [58]. 

The coherence of our anti-CD31 antibody results, which is a more specific endothelial marker, will enhance our understanding of the differences in vasculature distribution across different regions in the SN region. In a study in the mouse cerebellum, authors reported that the gray matter (cerebellar cortex) had a relatively short but dense network of microvessels (stained using anti-laminin antibody) with a short diffusion distance, whilst the white matter had a longer, less dense microvasculature with a greater diffusion distance [60], thereby highlighting that the character of the vascular bed corresponds with the demand for nutrients and blood supply. Similar findings were reported by Schnieder et al., who reported approximately 60% more vascular surface area density in both ventral and dorsal gray matter than white matter in the human brain (stained using anti-GLUT-1 antibody) [61]. Additionally, we found a higher CD31+ vessel density in the SNpc than the SNpr (Table 2), which depicts the regional vulnerability to vessel loss in aging, as reviewed by Pandya and Patani [45].

### 4.2. Alcohol Use Aggravates Decreased Microvascular Density in Both Gray and White Matter

Aging has long been considered as a potent factor influencing the density of the microvessels in brain and other tissues [62,63]. In a study in adult rats, Villar-Cheda et al. demonstrated that aged and sedentary low-exercising rats showed significant decreases in SN vascular density, indicating an age-related progressive decline in the functional and structural integrity of the SN region coupled with increased vulnerability to injury [64]. In our study, we found that aging plays a crucial role in regulating the density of CD31+ vessels. We noted significant differences between chronic alcoholics and both controls and age-matched alcoholics (groups A–C and groups B and C, respectively; Figure 2), thereby showing the possible effects of aging on vascular density. However, this decline in vasculature density was also mediated by the effects of alcohol, as evident from the significant differences obtained between controls and age-matched alcoholics (groups A and B; Figure 2). In previous studies, it has been shown that vascular density decreases with age in the human brain and is universally seen across all regions of the brain, although the rates of decrease may not be uniform [60,65,66,67,68]. Apart from the physiological aging-related decrease in the microvascular density, chronic ethanol intoxication has also been demonstrated to cause accelerated reductions in the terminal vascularization density due to disturbances in the angiogenesis [68]. 

CD31 is known to play a cytoprotective role in the endothelium [69]. Our findings on reduced expression of CD31 in alcoholics (Table 2; Figure 2) might indicate the increased vascular stress in the microvascular bed. Furthermore, in a study in human angiosarcoma samples, Venkataramani et al. demonstrated that the downregulation of CD31 expression led to loss of endothelial tube formation and an increased induction of antioxidative enzymes [70]. In addition, it has been shown that in PD patients, apart from a decline in the vascular density, there is a morphological transformation in the vessel structure. The vasculature becomes less branched along with the formation of endothelial cell “clusters”, which may be formed due to capillary fragmentation [71]. The authors described a “ladder-like” effect with areas of absent staining followed by areas of clustered staining (as seen in Figure 2) [71].

### 4.3. Gray Matter Has Thinner and More Damaged Collagen-Iv-Containing Basement Membrane Than White Matter

Our results indicate that there was a more global expression of collagen-IV coupled with smoother and more preserved integrity in the white matter vasculature than in the gray matter in both the SNpc and SNpr in controls (Table 3 and Table 4). Animal studies have shown that the gray matter is more rigid, stiff, and fragile than the surrounding white matter [72,73] and accumulates with aging. Although this difference in rigidity comes from a multitude of factors, including physical cell–cell interactions, decreased collagen-IV thickness or expression per vessel in the microvasculature of the gray matter plays a contributing role in determining the overall tissue viscoelasticity [74]. 

Previous studies have quantified collagen-IV immuno-stained capillaries in various regions of the brain [30,32,75]. In a recent study, Hase et al. analyzed the capillary width using anti-collagen-IV antibody in the frontal cortex and the underlying white matter. The authors found that microvessels in the white matter in controls were significantly wider (or dilated) and were significantly more immunostained than the microvessels in the gray matter [51]. Our observations are consistent with the results obtained by these authors (Figure 3). Relative quantification of collagen-IV concentrations using ELISA showed higher protein concentrations in the deep gray matter, brainstem, and cerebellum as compared to white matter regions such as the *corona radiata* and *corpus callosum* [76]. Another study, however, reported no significant differences in collagen distribution between gray and white matter regions using Masson’s trichrome staining [77]. 

We postulate that it is possible that the total content of collagen-IV may be higher in the gray matter due to a higher density of the microvasculature, although the distribution per vessel favors the white matter where the larger vessels have thicker BMs. Additionally, it is worth pointing out that in the previous studies the control samples studied represented older populations (55–92 years) as compared to the relatively younger controls (median age 31 years) we investigated in the present study. Studies in rat models have shown that the collagen-IV content decreases with aging and maturation, whilst the stiffness of the microvessels increases, showing the structural alterations the BM of the microvasculature undergoes [73,78,79]. 

Uspenskaia et al. showed that aging is associated with increased collagen type IV accumulation in the basal lamina of human cerebral microvessels [75]. Contrary to this, Rubio-Araiz et al. showed that in the post-mortem brain tissue samples obtained from the pre-frontal cortex of alcoholics, there were significant reductions in the immunoexpression of laminin and collagen-IV [80]. Using TEM, we observed at the ultrastructural level that the BM is multi-lamellar or split (Figure 6; Supplementary Table S4), which could potentially explain the differences noted by the previous authors, whereby a reduction in collagen-IV levels was noted. Furthermore, the differences in the vascular structure, spatial organization, and role and contribution of the string and coiled vessels towards the distribution and quantification of collagen-IV in the gray and white matter can explain the differences obtained in previous studies and ours. 

### 4.4. The Dual Role of Upregulated Expression in Laminin-111 

As a major non-collagen protein component of the BM, laminins play a critical role in promoting endothelial differentiation and BBB stability, as demonstrated by rat knockout models showing complete BBB leakage to outright hemorrhage [22]. In fact, depletion of astrocytic laminin-111 leads to disturbances in the differentiation of pericytes, which negatively affects the BBB’s integrity [81]. In agreement with our findings on increased laminin-111 expression, especially in chronic alcoholics (Table 5 and Table 6), it has been demonstrated that in chronic mild hypoxia, upregulation of laminin-111 occurs, which bolsters the vascular structural and functional integrity against the insults [82]. Similar upregulated expression has also been reported in mice models who controlled blunt head trauma [83]. The authors found that the circumference of laminin-enriched vessels was surrounded by astrocytic processes, thereby continuously secreting laminin to seal the breaches in the BBB, restructure the basal lamina, and ultimately restore the BBB’s integrity [83]. 

Apart from restoration of the BBB’s integrity, laminins have also been shown to confer neuroprotection to neural cells. Increased expression of both laminins and collagen has been shown to reduce Aβ secretion, thereby playing a neuroprotective role in AD [84]. Emerging evidence also points to a possible interaction between laminins and microglia. It has been shown that laminins not only act as anchoring structures for microglia, but also act as “food” for microglial cells in order for these cells to maintain an amoeboid morphology [85]. Furthermore, laminins have been shown to induce a pro-inflammatory phenotype in the microglia (lower cell volume and ramification) and to promote neuroinflammation [86]. These facts correlate with our previous findings in the same cohort of individuals, whereby we showed a dystrophic microglial phenotype and migration of perivascular microglia to diffuse locations in alcoholics [46]. This migration of microglia can explain the increased accumulation of laminins in the BM, since there is an imbalance in the consumption–production ratio.

### 4.5. Increased Expression of Fibronectin Might Promote Endothelial Damage

Fibronectin is largely absent in healthy adult brain tissue but is found abundantly during brain development [83]. During brain injury, the levels of fibronectin are known to increase, especially those of the soluble form, which possibly act as opsonins and aid macrophage clearance of the dead tissue and cellular debris [83]. An increased expression of fibronectin has been reported in post-mortem brain samples from both the frontal and temporal cortex in AD patients [87]. Evidence of an increase in the expression of fibronectin in stroke and a contrary decrease in expression in AD was reviewed by Thomsen et al. [20].

In our study, we found that alcoholics showed a sustained increase in fibronectin expression, indicating alcohol-mediated structural changes in the BBB. Furthermore, Nakakura et al. demonstrated that fibronectin is essential for the formation of fenestrae in the endothelial cells of the fenestrated capillaries [88]. These transcellular pores characterize the fenestrated endothelium found in the liver, kidneys, and different endocrine glands and aids in increasing the exchange of solutes. In contrast, the lack of fenestrae in the continuous endothelium of the BBB provides a strict endothelial control in a paracellular manner [89]. Our ultrastructural findings indicate alterations in the endothelial cell structure, thereby damaging the BBB (Figure 7). Such changes may be attributable to the increased amount of fibronectin in the vascular BM (Table 7 and Table 8). However, the exact mechanisms of how fibronectin interacts with other ECM components in the context of alcoholism remain largely unknown, and future studies are needed to shed light on the neuroprotective role of fibronectin.

Finally, from the results and discussion presented above, it is evident that the imbalance in the ratio between synthesis–consumption rates of the BM proteins led to deposition and overexpression of ECM proteins in alcoholics. It has been suggested that this imbalance is astrocyte-mediated, whereby astrocytes presumably goes into “overdrive” mode (possibly in conjunction with pericytes) in response to BBB injury to quickly repair the BBB integrity and structure [20,90]. However, emerging evidence from other authors and from our previous study suggests a possible role for microglia in controlling the consumption part of the equation. It is intriguing that whilst on the one hand we saw an increased thickness or expression of ECM proteins, on the other hand we saw it to be associated with a more lamellar and discontinuous BM (Figure 6). This potentially could indicate two-pronged stimulation of astrocytes—one process driving the synthesis of ECM proteins, with the other driving the release of MMPs and other microglial ECM-degrading enzymes. This could explain the “ladder-like clustered” vessel appearance seen in the present study and by previous authors.

### 4.6. Potential Diagnostic Applications

The results from the present study provide interesting insights into the vascular responses to alcohol exposure both without the effect of aging and with the aging. Changes in the vascular component of the blood–brain barrier can be quantified and assessed clinically using BOLD (blood oxygenation level-dependent) signals in fMRI (functional magnetic resonance imaging) [91]. Changes in endothelial function and increased thickness of the vessel wall due to collagen-IV, laminin-111, and fibronectin deposition would lead to decreased lumen in the vessel, along with increased viscoelasticity, which would affect the CVR (cerebrovascular reactivity) measurements in the fMRI [91,92]. Additionally, since we noted a global decrease in CD31+ vessels in the SN of alcoholics, such changes could reduce the rCBF (resting cerebral blood flow) when measured using doppler ultrasonography, radiotracer techniques, and phase contrast imaging [93,94,95]. Positron emission tomography can also be used for these purposes. A more detailed methodology for evaluating changes in vasculature clinically has been discussed elsewhere [91,92,93,94,95]. Since the present study was conducted on brain autopsy material, we cannot provide an exact clinical correlation of the observed changes in the present study with clinical parameters. Future studies to investigate this aspect are needed.

### 4.7. Limitations of the Present Study

The results from the present study are nevertheless constrained by some limitations, which need to be discussed. Firstly, the number of investigated individuals per group was relatively low, and our study included only samples from male individuals in the control group (group A), whilst the alcoholics groups (groups B and C) had a mix of samples from both male and female individuals. This could have introduced some underlying gender bias in our results. However, we would like to point out that upon exclusion of the female individuals from groups B and C (i.e., only male individuals included), we found similar tendencies and trends as reported in the results section of the study. Future studies with larger and more diverse groups are needed to encapsulate the true effects of gender regarding changes in the BM composition and structure. 

Secondly, more immunomarkers need to be explored, including junctional proteins such as claudins and occludins, as well as ECM component proteins such as nidogen and heparin sulphates, in order to understand the effects on the BBB as a whole. Additionally, the role of vascular growth or modeling enzymes, including matrix metalloproteinases (MMPs) and vascular endothelial growth factor (VEGF), needs to be investigated. Thirdly, the functional morphologies of pericytes and astrocytes need to be examined in future studies to understand the effects of aging, alcohol, and oxidative stress on these cells. Finally, the results we obtained in the present study could not be quantified in absolute protein terms (due to the small tissue sample). Our observations could be affected by the differences in sectioning of the tissue material or 2D observational biases. However, it is important to highlight that we investigated a large enough number of vessels (approximately 8000–10,000 vessels per immunomarker) using two independent observers and presented the results in terms of weighted averages (thereby eliminating bias), which should bring the margin of error within acceptable limits.

## 5. Conclusions

In the present study, we demonstrate that in physiological conditions, the white matter shows significantly fewer CD31+ vessels in comparison to the gray matter, with the microvascular density being significantly decreased in both SN regions in response to chronic alcohol exposure. Furthermore, increased expression of BM proteins (collagen-IV, laminin-111, and fibronectin) was noted in both age-matched and chronic alcoholics, which leads to the thickening of the vascular BM. Alcohol exposure at the same time leads to increased splitting and disruption of the vascular membrane continuity, as evident from visualization of fenestrae and pores in ultrastructural and structural analyses.

All of these aforementioned changes lead to the formation of clustered, ladder-like vessels characteristic of the region, thereby contributing towards the dysregulation of the local tissue environment. Future studies with larger cohorts investigating other aspects of the BBB, including cellular responses, are needed to fully encapsulate the effects of aging and alcoholism on composition and structure of the vascular BM. 

## Figures and Tables

**Table 1:** Primary antibodies used in the present study.

Primary Antibody *	Antibody Properties **	Clone	Working Dilutions	Manufacturer	Catalogue No.
CD31	Mouse monoclonal AB against human AG	JC70A	1/30	DakoCytomation (Glostrup, Denmark)	M0823
Collagen-IV	Mouse monoclonal AB against human AG	PHM-12	1/100	Novocastra (Deer Park, IL, USA)	NCL-COLL-IV
Laminin-111	Rabbit polyclonal AB against human AG	-	1/1000	Arigo Biolaboratories (Hsinchu City, Taiwan)	ARG10736
Fibronectin	Rabbit Polyclonal AB against human AG	-	1/400	DakoCytomation (Glostrup, Denmark)	A0245

* CD31—cluster of differentiation 31; ** AB, antibody; AG, antigen.

**Table 2:** Distribution of CD31+ vessels per visual field in different regions of SN.

	Controls (Group A)	Young Alcoholics (Group B)	Chronic Alcoholics (Group C)	*p* Value ^†^
***Pars Compacta*** **(SNpc)**				
Gray Matter	07.00 ± 0.17	05.80 ± 0.14	05.03 ± 0.09	<0.001 **
White Matter	05.09 ± 0.14	03.83 ± 0.11	03.34 ± 0.07	<0.001 **
***p*** **Value ^‡^**	<0.001 **	<0.001 **	<0.001 **	-
***Pars Reticulata*** **(SNpr)**				
Gray Matter	05.55 ± 0.18	04.26 ± 0.16	03.52 ± 0.10	<0.001 **
White Matter	04.34 ± 0.12	02.56 ± 0.17	02.02 ± 0.08	<0.001 **
***p*** **Value ^‡^**	<0.001 **	<0.001 **	<0.001 **	-

Note: ^†^ *p* value was calculated for Kruskal–Wallis ANOVA (inter-group analysis); ^‡^ *p* value was calculated for related-samples Wilcoxon signed rank test (intra-group analysis). The numbers represent the average number of CD31+ vessels per visual field ± S.E. (standard error); ** indicates a significant difference between groups (*p* < 0.05 is considered as significant with Bonferroni correction for Kruskal–Wallis ANOVA and without correction for related-samples Wilcoxon signed rank test).

**Table 3:** Inter-group and intra-group comparisons of the differences in the grades of thickness based on the expression of collagen-IV in the BM of microvessels in the SN region (shown by weighted average).

	Controls (Group A)	Young Alcoholics (Group B)	Chronic Alcoholics (Group C)	*p* Value ^†^
***Pars Compacta*** **(SNpc)**				
Gray Matter	1.084	1.102	1.195	<0.001 **
White Matter	1.121	1.143	1.187	0.009 **
***p*** **Value ^‡^**	0.072	0.031 **	0.355	-
***Pars Reticulata*** **(SNpr)**				
Gray Matter	1.063	1.130	1.158	<0.001 **
White Matter	1.115	1.160	1.201	0.002 **
***p*** **Value ^‡^**	0.002 **	0.991	0.187	-

Note: ^†^ *p* value was calculated for Kruskal–Wallis ANOVA (inter-group analysis); ^‡^ *p* value was calculated for related-samples Wilcoxon signed rank test (intra-group analysis); ** indicates a significant difference between groups (*p* < 0.05 is considered as significant with Bonferroni correction for Kruskal–Wallis ANOVA and without correction for related-samples Wilcoxon signed rank test).

**Table 4:** Inter-group and intra-group comparisons of the differences in the grades of integrity based on the expression of collagen-IV in the BM of microvessels in the SN region (shown by weighted average).

	Controls (Group A)	Young Alcoholics (Group B)	Chronic Alcoholics (Group C)	*p* Value ^†^
***Pars Compacta*** **(SNpc)**				
Gray Matter	1.201	1.245	1.343	<0.001 **
White Matter	1.116	1.195	1.216	<0.001 **
***p*** **Value ^‡^**	<0.001 **	0.004 **	<0.001 **	-
***Pars Reticulata*** **(SNpr)**				
Gray Matter	1.173	1.209	1.241	0.012 **
White Matter	1.121	1.216	1.296	<0.001 **
***p*** **Value ^‡^**	<0.001 **	0.939	0.452	-

Note: ^†^ *p* value was calculated for Kruskal–Wallis ANOVA (inter-group analysis); ^‡^ *p* value was calculated for related-samples Wilcoxon signed rank test (intra-group analysis); ** indicates a significant difference between groups (*p* < 0.05 is considered as significant with Bonferroni correction for Kruskal–Wallis ANOVA and without correction for related-samples Wilcoxon signed rank test).

**Table 5:** Inter-group and intra-group comparisons of the differences in the grades of thickness of the BM based on the expression of laminin-111 in microvessels in the SN region (shown by weighted average).

	Controls (Group A)	Young Alcoholics (Group B)	Chronic Alcoholics (Group C)	*p* Value ^†^
***Pars Compacta*** **(SNpc)**				
Gray Matter	1.132	1.192	1.249	<0.001 **
White Matter	1.179	1.213	1.280	0.005 **
***p*** **Value ^‡^**	0.146	0.949	0.611	-
***Pars Reticulata*** **(SNpr)**				
Gray Matter	1.163	1.226	1.295	<0.001 **
White Matter	1.188	1.257	1.344	<0.001 **
***p*** **Value ^‡^**	0.333	0.786	0.279	-

Note: ^†^ *p* value was calculated for Kruskal–Wallis ANOVA (inter-group analysis); ^‡^ *p* value was calculated for related-samples Wilcoxon signed rank test (intra-group analysis); ** indicates a significant difference between groups (*p* < 0.05 is considered as significant with Bonferroni correction for Kruskal–Wallis ANOVA and without correction for related-samples Wilcoxon signed rank test).

**Table 6:** Inter-group and intra-group comparisons of the differences in the grades of integrity of the BM based on the expression of laminin-111 in microvessels in the SN region (shown by weighted average).

	Controls (Group A)	Young Alcoholics (Group B)	Chronic Alcoholics (Group C)	*p* Value ^†^
***Pars Compacta*** **(SNpc)**				
Gray Matter	1.214	1.219	1.396	<0.001 **
White Matter	1.169	1.213	1.360	0.003 **
***p*** **Value ^‡^**	0.011 **	0.936	0.830	-
***Pars Reticulata*** **(SNpr)**				
Gray Matter	1.176	1.251	1.326	<0.001 **
White Matter	1.174	1.227	1.318	<0.001 **
***p*** **Value ^‡^**	0.428	0.482	0.526	-

Note: ^†^ *p* value was calculated for Kruskal–Wallis ANOVA (inter-group analysis); ^‡^ *p* value was calculated for related-samples Wilcoxon signed rank test (intra-group analysis); ** indicates a significant difference between groups (*p* < 0.05 is considered as significant with Bonferroni correction for Kruskal–Wallis ANOVA and without correction for related-samples Wilcoxon signed rank test).

**Table 7:** Inter-group and intra-group comparisons of the differences in the grades of thickness based on the expression of fibronectin in the BM of the microvessels in the SN region (shown by weighted average).

	Controls (Group A)	Young Alcoholics (Group B)	Chronic Alcoholics (Group C)	*p* Value ^†^
***Pars Compacta*** **(SNpc)**				
Gray Matter	1.145	1.158	1.217	<0.001 **
White Matter	1.185	1.317	1.380	<0.001 **
***p*** **Value ^‡^**	0.010 **	<0.001 **	0.033 **	-
***Pars Reticulata*** **(SNpr)**				
Gray Matter	1.122	1.159	1.210	<0.001 **
White Matter	1.162	1.240	1.339	<0.001 **
***p*** **Value ^‡^**	0.923	0.002 **	0.026 **	-

Note: ^†^ *p* value was calculated for Kruskal–Wallis ANOVA (inter-group analysis); ^‡^ *p* value was calculated for related-samples Wilcoxon signed rank test (intra-group analysis); ** indicates a significant difference between groups (*p* < 0.05 is considered as significant with Bonferroni correction for Kruskal–Wallis ANOVA and without correction for related-samples Wilcoxon signed rank test).

**Table 8:** Inter-group and intra-group comparisons of the differences in the grades of integrity based on expression of fibronectin in the BM of the microvessels in the SN region (shown by weighted average).

	Controls (Group A)	Young Alcoholics (Group B)	Chronic Alcoholics (Group C)	*p* Value ^†^
***Pars Compacta*** **(SNpc)**				
Gray Matter	1.132	1.135	1.248	<0.001 **
White Matter	1.082	1.215	1.298	<0.001 **
***p*** **Value ^‡^**	<0.001 **	0.063	<0.001 **	-
***Pars Reticulata*** **(SNpr)**				
Gray Matter	1.158	1.181	1.261	<0.001 **
White Matter	1.097	1.173	1.247	0.273
***p*** **Value ^‡^**	<0.001 **	0.086	<0.001 **	-

Note: ^†^ *p* value was calculated for Kruskal–Wallis ANOVA (inter-group analysis); ^‡^ *p* value was calculated for related-samples Wilcoxon signed rank test (intra-group analysis); ** indicates a significant difference between groups (*p* < 0.05 is considered as significant with Bonferroni correction for Kruskal–Wallis ANOVA and without correction for related-samples Wilcoxon signed rank test).

## Data Availability

All the data used in this study are available from the corresponding author upon request.

## Supplementary Materials

The following supporting information can be downloaded at: https://www.mdpi.com/article/10.3390/biomedicines10040830/s1: Table S1: Characteristics of the brain autopsy individuals. Table S2: Frequencies (%) showing the distribution of vessels based on the semi-quantitative grading scale for thickness in different regions of *substantia nigra* (SN). Table S3: Frequencies (%) showing the distribution of vessels based on the semi-quantitative grading scale for integrity in different regions of *substantia nigra* (SN). Table S4: Table of measurements for the thickness of the basement membrane of the microvessels (in nm). Figure S1: Corrected total cell fluorescence (CTCF) depicting the immunofluorescence signal intensity for the three-basement membrane proteins. Figure S2: Negative (phosphate-buffered saline—PBS) controls for immunohistochemistry (IHC) and immunofluorescence (IF) reactions in the SN region of the brain.
