# Supplementary material for: Alcohol-Induced Alterations in the Vascular Basement Membrane in the Substantia Nigra of the Adult Human Brain
Source: Biomedicines. 2022 Apr 1;10(4):830. doi: 10.3390/biomedicines10040830 (PMC9028457; doi:10.3390/biomedicines10040830)
Supplement: Supplementary file 1 [file biomedicines-10-00830-s001.zip › biomedicines-1602202-supplementary.pdf]

SUPPLEMENTARY MATERIAL

Alcohol-Induced Alterations in the Vascular Basement Membrane in the *Substantia Nigra* of Adult Human Brain

Sandra Skuja, Nityanand Jain, Marks Smirnovs, and Modra Murovska

Index

1. **Supplementary Table S1.** Characteristics of brain autopsy individuals.

2. **Supplementary Table S2.** Frequencies (%) showing the distribution of vessels based on the semi-quantitative grading scale for thickness in different regions of Substantia Nigra (SN).

3. **Supplementary Table S3.** Frequencies (%) showing the distribution of vessels based on the semi-quantitative gradingscale for integrity in different regions of *Substantia Nigra* (SN).

4. **Supplementary Table S4.** Table of measurements for the thickness of the basement membrane (BM) of the microvessels (in nm).

5. **Supplementary Figure S1.** Corrected total cell fluorescence (CTCF) depicting the immunofluorescence signal intensity for the three basement membrane proteins.

6. **Supplementary Figure S2.** Negative (phosphate-buffer saline - PBS) controls for immunohistochemistry (IHC) and immunofluorescence (IF) reactions in the SN region of the brain.

Supplementary Table S1. Characteristics of the brain autopsy individuals.

Group A (Control Group)			Group B (Young Alcoholics)			Group C (Chronic Alcoholics)		
**Individual Code**	**Age (years)**	**Gender**	**Individual Code**	**Age (years)**	**Gender**	**Individual Code**	**Age (years)**	**Gender**
A1	34	M	B1	36	F	C1	48	M
A2	31	M	B2	23	M	C2	55	M
A3	27	M	B3	31	M	C3	60	M
A4	23	M	B4	26	M	C4	50	F
A5	32	M	B5	33	M	C5	63	M
A6	37	M	B6	34	F	C6	45	M
A7	33	M	B7	25	F	C7	45	M
A8	22	M	B8	30	M	C8	55	M
A9	36	M	B9	35	M	C9	45	F
A10	17	M	B10	22	M	C10	60	F
A11	37	M	B11	35	M	C11	66	M
A12	20	M	B12	34	M	C12	63	M
A13	26	M	B13	29	M	C13	44	F
						C14	49	M
						C15	45	F
						C16	60	M
						C17	38	F
						C18	40	M
**Group Median Age**		31 ± 6.79	**Group Median Age**		31 ± 4.85	**Group Median Age**		49.5 ± 8.66

Supplementary Table S2. Frequencies (%) showing the distribution of vessels based on the semi-quantitative grading scale for thickness in different regions of *Substantia Nigra* (SN).

Region	Group	Grade I (%)	Grade II (%)	Grade III (%)
Collagen-IV				
SNpc Gray Matter	A	91.90	07.80	00.30
	B	90.30	09.20	00.50
	C	81.20	18.10	00.70
SNpc White Matter	A	87.94	12.06	00.00
	B	85.82	13.99	00.19
	C	82.04	17.25	00.72
SNpr Gray Matter	A	93.75	06.25	00.00
	B	87.06	12.89	00.05
	C	84.26	15.67	00.07
SNpr White Matter	A	88.55	11.45	00.00
	B	85.13	13.75	01.12
	C	81.16	17.04	01.80
Laminin-111				
SNpc Gray Matter	A	86.94	12.96	00.10
	B	82.64	15.52	01.85
	C	79.07	16.99	03.95
SNpc White Matter	A	82.07	17.93	00.00
	B	79.78	19.13	01.09
	C	74.89	22.21	02.91
SNpr Gray Matter	A	83.70	16.30	00.00
	B	79.27	18.79	01.94
	C	74.64	21.20	04.16
SNpr White Matter	A	81.48	18.29	00.23
	B	75.84	22.68	01.49
	C	69.87	25.90	04.23
Fibronectin				
SNpc Gray Matter	A	88.55	11.45	00.00
	B	85.12	13.90	00.98
	C	80.74	16.78	02.47
SNpc White Matter	A	81.78	17.84	00.38
	B	71.43	25.37	03.20
	C	66.87	28.19	04.95
SNpr Gray Matter	A	87.96	11.90	00.13
	B	85.08	13.87	01.05
	C	81.49	16.05	02.45
SNpr White Matter	A	83.79	16.21	00.00
	B	77.82	20.30	01.88
	C	71.09	23.87	05.04

Abbreviations: SNpc: *Substantia Nigra pars compacta;* SNpr: *Substantia Nigra pars reticulata.* Group A – controls, Group B – young alcoholics and Group C – chronic alcoholics.

Supplementary Table S3. Frequencies (%) showing the distribution of vessels based on the semi-quantitative grading scale for integrity in different regions of *Substantia Nigra* (SN).

Region	Group	Grade I (%)	Grade II (%)	Grade III (%)
Collagen-IV				
SNpc Gray Matter	A	81.60	16.70	01.70
	B	77.40	20.70	01.90
	C	71.90	21.90	06.20
SNpc White Matter	A	88.93	10.47	00.59
	B	82.04	16.45	01.51
	C	80.72	17.01	02.28
SNpr Gray Matter	A	83.67	15.32	01.01
	B	80.76	17.58	01.66
	C	79.14	17.64	03.22
SNpr White Matter	A	88.12	11.66	00.22
	B	80.30	17.84	01.86
	C	75.07	20.22	04.71
Laminin-111				
SNpc Gray Matter	A	78.58	21.42	00.00
	B	78.20	21.67	00.12
	C	62.56	35.27	02.17
SNpc White Matter	A	83.07	16.93	00.00
	B	78.70	21.30	00.00
	C	65.70	32.62	01.68
SNpr Gray Matter	A	82.40	17.60	00.00
	B	75.16	24.62	00.22
	C	68.34	30.69	00.97
SNpr White Matter	A	82.64	17.36	00.00
	B	77.70	21.93	00.37
	C	69.22	29.80	00.98
Fibronectin				
SNpc Gray Matter	A	86.64	13.36	00.00
	B	86.51	13.49	00.00
	C	75.56	24.05	00.39
SNpc White Matter	A	92.22	07.40	00.38
	B	81.88	14.71	03.41
	C	74.36	21.44	04.20
SNpr Gray Matter	A	84.13	15.87	00.00
	B	82.56	16.81	00.63
	C	76.07	21.78	02.15
SNpr White Matter	A	90.27	09.73	00.00
	B	83.46	15.79	00.75
	C	77.98	19.33	02.69

Abbreviations: SNpc: *Substantia Nigra pars compacta;* SNpr: *Substantia Nigra pars reticulata.* Group A – controls, Group B – young alcoholics and Group C – chronic alcoholics.

Supplementary Table S4. Table of measurements for the thickness of the basement membrane (BM) of the microvessels (in nm).

Vessel No.	No. of observations	Grade of Vessel*	Average thickness of separate lamella**	Minimum thickness of basement membrane	Maximum thickness of basement membrane
1	10	II	170.33 ± 82.47	68.62	355.67
2	10	II	209.05 ± 51.39	131.12	285.88
3	10	II	212.80 ± 97.92	102.77	353.00
4	10	I	97.86 ± 46.83	39.42	195.34
5	10	II	210.09 ± 94.38	118.50	409.36
6	10	III	967.66 ± 143.72	753.88	1233.89
7	10	III	544.43 ± 222.28	137.46	778.18
8	10	II	168.77 ± 103.67	67.97	423.66
9	10	II	183.92 ± 86.84	80.00	339.93
10	10	II	220.06 ± 116.80	92.21	459.40
11	10	II	250.88 ± 194.12	55.10	608.31
12	10	III	312.26 ± 266.77	82.46	777.93
13	10	I	107.73 ± 47.13	39.60	164.78
14	10	II	206.13 ± 120.61	67.42	424.00
15	10	III	291.73 ± 241.53	82.09	887.96
16	10	III	633.50 ± 723.24	120.87	2406.77
17	10	II	197.81 ± 135.68	45.17	416.48
18	10	II	194.13 ± 160.46	54.61	516.61
19	10	II	240.65 ± 97.92	83.67	344.36
20	10	III	225.21 ± 223.48	80.00	821.52
21	10	II	172.21 ± 73.03	60.00	286.34
22	10	II	244.78 ± 126.42	52.36	404.90
23	10	III	251.11 ± 255.92	50.49	723.04
24	10	III	245.03 ± 205.14	52.55	752.91
25	10	II	204.36 ± 131.43	29.80	480.33
26	10	II	238.76 ± 166.69	39.22	557.36
27	10	II	242.49 ± 68.52	151.44	381.23
28	10	II	208.97 ± 135.58	48.70	449.70
29	10	I	115.90 ± 38.43	39.73	158.94
30	10	II	126.69 ± 75.88	38.87	275.98
	**Average ****		256.54 ± 173.31 nm	95.54 ± 128.54 nm	555.79 ± 424.61 nm

* Cumulative grade of the vessel in terms of both integrity and thickness; Grade I vessel defined as maximum thickness from 20-200 nm with smooth and homogenous basement membrane; Grade II vessel defined as maximum thickness from 201-700 nm with lamellar basement membrane and Grade III vessel defined as maximum thickness greater than 701 nm with split basement membrane. These gradings were created based on TEM observations to correlate them with the semi-quantitative scoring scale that was used for immunohistochemical analysis (refer to Figure 1). ** Average thickness ± standard deviation (S.D.).

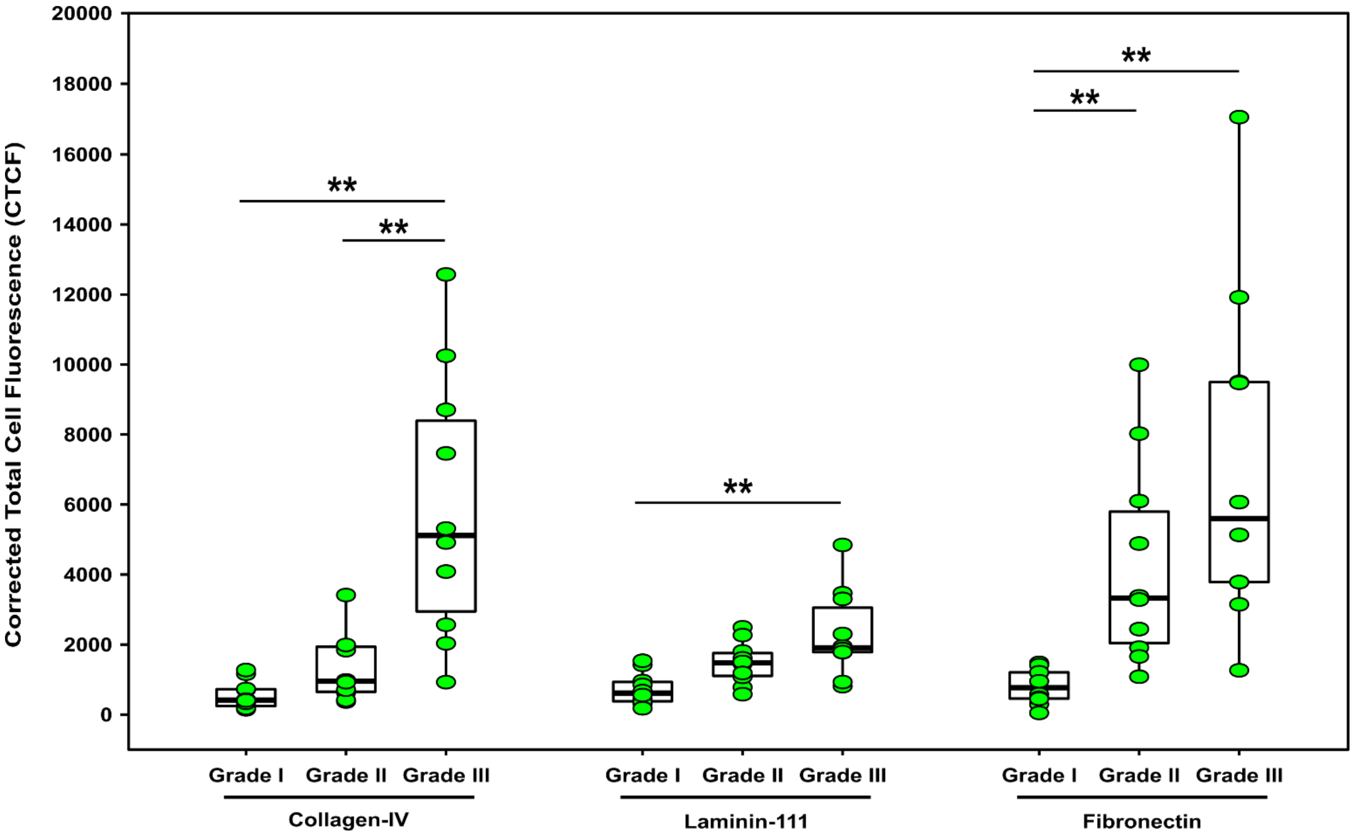

Supplementary Figure S1. Corrected total cell fluorescence (CTCF) depicting the immunofluorescence signal intensity for the three basement membrane proteins. ** indicates a significant difference between the groups ($P < 0.05$ with Bonferroni correction is considered as significant; Related-samples Friedman's two-way ANOVA). Grade I vessel represents normal (baseline) thickness vessel with unchanged integrity; Grade II vessel represents moderately thickened vessel with damaged integrity and Grade III vessel represents extremely thickened vessel with split basement membrane.

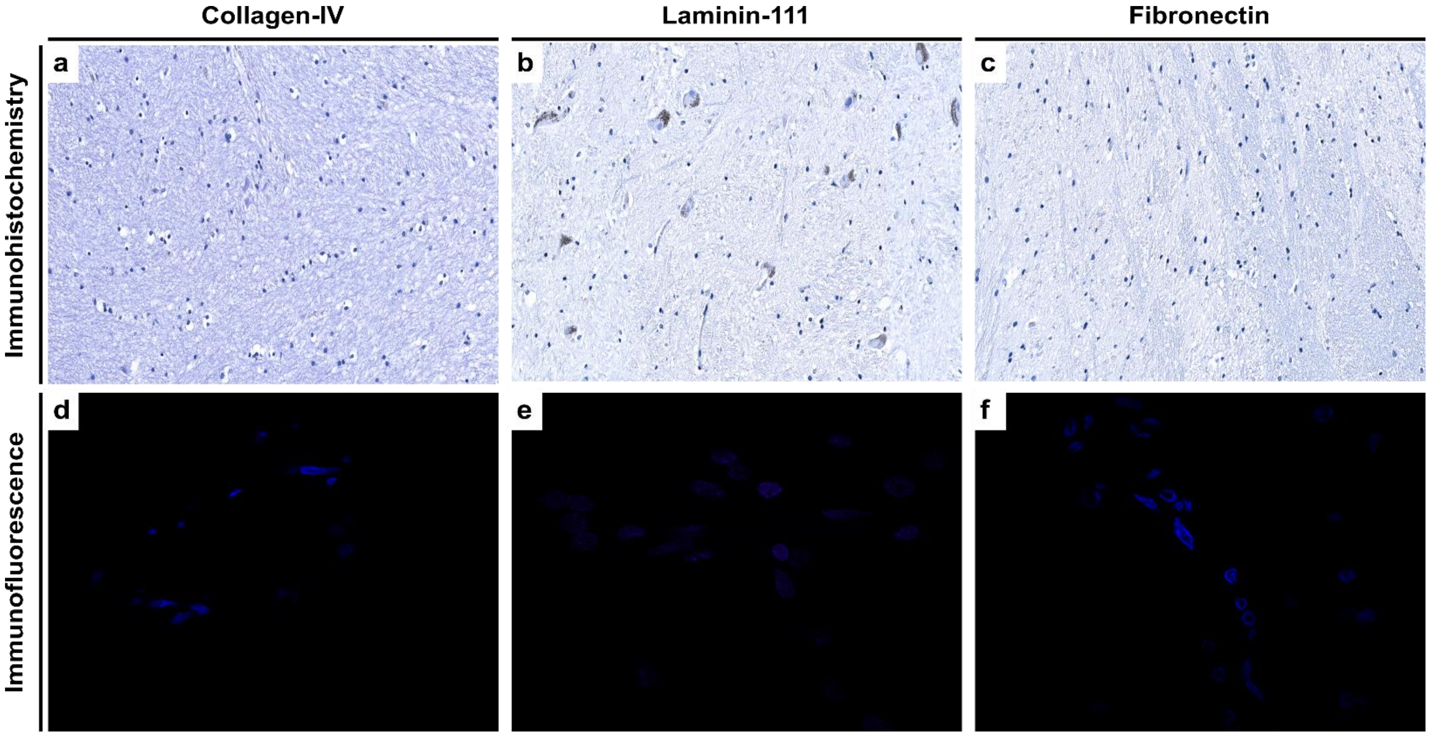

Supplementary Figure S2. Negative (phosphate-buffer saline - PBS) controls for (**a**,**b**,**c**) immunohistochemistry (IHC) and (**d**,**e**,**f**) immunofluorescence (IF) reactions in the SN region of the brain. In IF, blue color shows DAPI stained nuclei. Original magnification (IHC), 200×. Original magnification (IF), 1000×.
